# A macropinocytosis-related gene signature predicts the prognosis and immune microenvironment in hepatocellular carcinoma

**DOI:** 10.3389/fonc.2023.1143013

**Published:** 2023-03-30

**Authors:** Xinjiang Ding, Tao Yao, Xi Liu, Zhongwen Fan, Yuanxing Liu

**Affiliations:** ^1^ Division of Hepatobiliary and Pancreatic Surgery, Department of Surgery, First Affiliated Hospital, School of Medicine, Zhejiang University, Hangzhou, China; ^2^ College of Life Science, Zhejiang Chinese Medicine University, Hangzhou, China; ^3^ School of Medicine, Zhejiang University, Hangzhou, China

**Keywords:** macropinocytosis, hepatocellular carcinoma, prognosis, tumor microenvironment, survival

## Abstract

**Background:**

Available treatments for hepatocellular carcinoma (HCC), a common human malignancy with a low survival rate, remain unsatisfactory. Macropinocytosis (MPC), a type of endocytosis that involves the non-specific uptake of dissolved molecules, has been shown to contribute to HCC pathology; however, its biological mechanism remains unknown.

**Methods:**

The current study identified 27 macropinocytosis-related genes (MRGs) from 71 candidate genes using bioinformatics. The R software was used to create a prognostic signature model by filtering standardized mRNA expression data from HCC patients and using various methods to verify the reliability of the model and indicate immune activity.

**Results:**

The prognostic signature was constructed using seven MPC-related differentially expressed genes, *GSK3B*, *AXIN1*, *RAC1*, *KEAP1*, *EHD1*, *GRB2*, and *SNX5*, through LASSO Cox regression. The risk score was acquired from the expression of these genes and their corresponding coefficients. HCC patients in the discovery and validation cohorts were stratified, and the survival of low-risk score patients was improved in both cohorts. Time-dependent ROC analysis indicated that the model’s prediction reliability was the highest in the short term. Subsequent immunologic analysis, including KEGG, located the immune action pathway of the differentially expressed genes in the direction of the cancer pathway, etc. Immune infiltration and immune checkpoint tests provided valuable guidance for future follow-up experiments.

**Conclusion:**

A risk model with MRGs was constructed to effectively predict HCC patient prognoses and suggest changes in the immune microenvironment during the disease process. The findings should benefit the development of a prognostic stratification and treatment strategy for HCC.

## Introduction

Primary liver cancer is a common malignancy associated with high mortality, with hepatocellular carcinoma (HCC) accounting for 85% to 90% of cases. HCC is the fourth most frequent cause of tumor grade and the second leading cause of all cancer-related deaths worldwide (1). The traditional treatments for HCC include surgery, radiofrequency ablation, chemotherapy, and liver transplantation, but their efficacy is limited. In recent years, significant progress in the development of targeted molecular therapies and immunotherapeutic agents has improved HCC patient survival (2). However, these treatment options are often impacted by tumor heterogeneity and may lead to the occurrence of drug resistance (3, 4). Thus, there is a need for strategies that effectively predict treatment prognosis. This would aid in the development of more targeted treatments and inform the need for adjuvant therapies to prevent tumor recurrence and metastasis (5).

Recent advances in gene sequencing technology have exposed the genetic makeup of multiple diseases, including HCC, by identifying diverse genes and pathways involved in different pathological processes, signal transduction pathways, and nutrient absorption processes. Macropinocytosis (MPC) is an actin-driven endocytic process that is biologically distinct from other types of endocytosis, such as clathrin-mediated (6). The dysregulation of MPC is involved in immune responses, tumor progression, and cell death during various diseases, including HCC (7). Tumor growth relies on nutrients such as glucose and glutamine (Gln). When they become limited in the tumor microenvironment, tumor cells use MPC to scavenge extracellular nutrients to support their growth (8). Reusing proteins extracted from the extracellular microenvironment establishes tumor cell superiority by bypassing *de novo* synthesis, which is most common when tumors are facing nutritional deficiencies (9). Tumor cells also use MPC to obtain necrotic cell residue and tissue debris. Recent studies have shown that reusing carbohydrates, lipids, and nucleotides from cell debris is an important method of acquiring nutrients (10, 11). As a result, it is important to identify MPC-related genes (MRGs) and analyze their functions during HCC. MPC and its molecular signaling pathways are shown to play a critical role in the development of cancer (12). Cancer cells expressing MRGs under nutrient deficiency stress can use MPC to induce tumor growth. Meanwhile, dysregulation of MPC and specified drug interventions can induce methuosis, a unique form of non-apoptotic cell death (13). However, the biological mechanism of MPC during HCC remains unclear. Thus, understanding the impact of MRGs on HCC patient survival is of considerable interest (14).

The current study compared MRG expression in normal and HCC tissue samples from public databases and used differentially expressed MRGs to construct a prediction signature model. In addition, immunity function enrichment and immune-related pathways were explored to discern the association between MPC and the tumor microenvironment and inform novel immunotherapeutic strategies for HCC (14).

## Materials and methods

### Public data collection

Genetic information and matched clinical parameters, including patient age, identification number, tumor clinical stage, and survival data from 40 normal and 374 HCC samples, were obtained from The Cancer Genome Atlas (TCGA) database (https://portal.gdc.cancer.gov/repository). Identity information and clinicopathological characteristics from 273 HCC samples were obtained as a validation cohort from the International Cancer Genome Consortium (ICGC) portal (https://dcc.icgc.org/releases/current/Projects/LIRI-JP). Furthermore, we got the identity information and clinicopathological characteristics of HCC samples from the International Cancer Genome Consortium (ICGC) portal (https://dcc.icgc.org/releases/current/Projects/LIRI-JP) as the main validation cohort and obtained gene expression data and corresponding clinicopathological features from the Gene Expression Omnibus (GEO) database (https://www.ncbi.nlm.nih.gov/geo/, ID: GSE14520) database for the secondary validation cohort. Before the subsequent analysis, we use the “sva” package of R to minimize the gene expression data batch effect between different databases and different batches.

We searched and obtained some relevant literature related to macropinocytosis from PubMed and manually extracted these 71 candidate MRGs. We attach the article catalog and 71 candidate MRGs in **Supplementary Document 1**.

### Identification of differentially expressed MRGs

The “limma” package of the Bioconductor in R software was used to compare the expression of MRGs from the TCGA between tumor and normal samples and to identify differentially expressed macropinocytosis-related genes (DEMRGs) (**Supplementary List 1**). DEMRGs with a *p*-value of <0.05 were considered significant.

### Construction and validation of a signature DEMRG prognostic model

The Least absolute shrinkage and selection operator (LASSO) Cox regression analysis with the “glmnet” package in R was used to screen for DEMRG expression and establish a prognostic model. Shrinkage of the regression coefficient (*β*) removed genes with a zero regression coefficient and reserved genes with a non-zero weight (15). The risk score of each HCC sample was determined using standardized HCC mRNA expression data (EXP) with a linear combination of the regression coefficients from the LASSO model. The risk score was the summation of the multiplied *β*s using DEMRG signal data [RS = ∑ EXP (mRNA) * coefficients], allowing an MPC-related prognostic model (MRPM) to be constructed. The HCC samples were divided into a low-risk score (LRS) and high-risk score (HRS) clusters using the med-cutoff RS with the “pheatmap” package in R. To determine whether samples in the two groups were well separated, principal component analysis (PCA) was conducted using the “ggplot2” package.

Independent risk factors and overall survival (OS) of the two groups were determined to evaluate the DEMRG forecasting value using univariate and multivariate Cox regression models and the log-rank test, respectively. A time-dependent receiver operating characteristic (ROC) curve was performed on each independent cohort to assess the ability of the MRPM to predict OS using the “time-ROC,” “survminer,” and “survival” packages (14). The ICGC and GEO cohorts were also used to verify the model in the same way to make it more convincing.

### HCC tissue specimens

Tissue samples, including 12 from HCC patients, were obtained from the First Affiliated Hospital of Zhejiang University School of Medicine after surgery, then quickly frozen and stored in −80°C freezers. All patients provided informed consent for their tissues to be used for research, and related procedures were conducted in accordance with the 2013 revised Declaration of Helsinki.

### Fluorescence quantitative real-time PCR

The RNA from the filtered HCC sample was extracted with TRIzol® reagent (15596018, Life Technologies) according to the manufacturer’s protocol. A spectrometer was used to detect the RNA concentration, the MultiScribeTM Reverse Transcriptase (4311235, Invitrogen) was used to carry out reverse transcription, and cDNA was used for the SYBR Green Master Mix (4367659, Applied BiosystemsTM) qRT-PCR. The 2−ΔΔCt method was used to determine the relative abundance of RNA by comparing the levels with a housekeeping gene such as *GADPH*. Related primer sequences are provided in **Supplementary Document 2**.

### Other statistics

A protein–protein interaction (PPI) network was created using the Search Tool for the Retrieval of Interacting Genes (STRING) Protein–Protein Interaction Networks Functional Enrichment Analysis (https://cn.string-db.org/), and a protein correlation network was plotted using the “igraph” and “reshape2” R packages to show interactions between the DEMRGs. Analysis of DEMRG immune function enrichment was evaluated using Gene Ontology (GO) with the “clusterProfiler” in R, and important biochemical relationships between the DEMGs were assessed using the Kyoto Encyclopedia of Genes and Genomes (KEGG). Single-sample gene set enrichment analysis (ssGSEA) was performed to determine the scores of infiltrating immunocytes and the activity of immunological pathways between the LRS and HRS groups using the “gsva” package (14). Kaplan–Meier survival curves and a log-rank test were performed to measure the predictive performance of the prognostic MRG signature (16).

All statistical analyses were performed using the R Project for Statistical Computing (version 4.1.2) and GraphPad Prism 9. Statistical significance was considered for data with a *p*-value <0.05, a false discovery rate (FDR) <0.05, and a |log2FoldChange| >1.

## Results

### DEMRG identification

**Figure 1:**
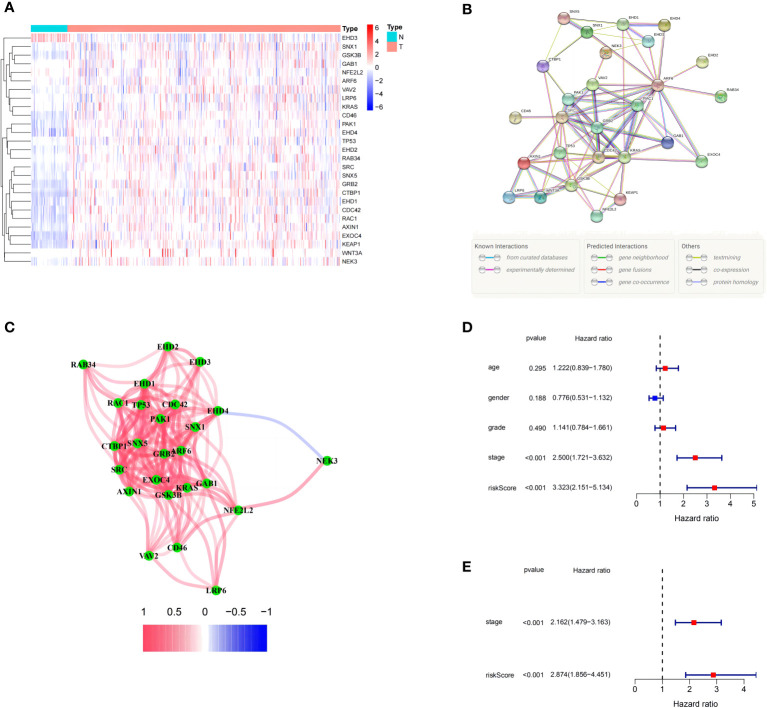
Expression of the 27 differentially expressed macropinocytosis-related genes (DEMRGs) and their interactions. **(A)** Heatmap (blue: low expression level; red: high expression level) of the DEMRGs between the non-tumor (N, blue) and tumor (T, red) samples. **(B)** The PPI network showing the interactions of the DEMRGs (interaction score = 0.4). **(C)** The correlation network of the DEMRGs (red line: positive correlation; blue line: negative correlation. The depth of the colors reflects the strength of the association). **(D, E)** Univariate and multivariate Cox regression analyses for the risk score. **(D)** Univariate analysis for the TCGA cohort. **(E)** Multivariate analysis for the TCGA cohort.

The expression levels of 71 MRGs were compared between normal and tumor samples from the TCGA. Using the established criteria, 27 DEMRGs were obtained, of which 25 (*CDC42*, *KRAS*, *ARF6*, *GSK3B*, *AXIN1*, *WNT3A*, *LRP6*, *PAK1*, *EXOC4*, *EHD2*, *RAC1*, *TP53*, *KEAP1*, *EHD1*, *EHD4*, *NEK3*, *SRC*, *GRB2*, *GAB1*, *CTBP1*, *RAB34*, *SNX1*, *SNX5*, *CD46*, and *VAV2*) were upregulated and two (*NFE2L2* and *EHD3*) were downregulated. The DEMRG expression levels are listed in **Figure 1A**. PPI networks were developed to determine the functional connectivity and interactions of the expressed proteins. The interaction score was set to 0.4 to obtain medium confidence (**Figure 1B**). *ARF6*, *RAC1*, *VAV2*, *PAK1*, *SRC*, GRB2, *KRAS*, and *CDC42* were defined as pivotal genes. All DEMRGs and their interaction network are shown in **Figure 1C**.

### Construction and validation of the signature DEMRG prognostic model

The HCC samples with intact survival information were obtained from the TCGA to assess the relationship between the DEMRGs and patient prognosis. There were 12 standard conforming genes, namely, *CDC42*, *PSD4*, *GSK3B*, *AXIN1*, *PAK1*, *RAC1*, *KEAP1*, *EHD1*, *SRC*, *GRB2*, *SNX5*, and *AKT1*, among which 11 genes, *CDC42*, *GSK3B*, *AXIN1*, *PAK1*, *RAC1*, *KEAP1*, *EHD1*, *SRC*, *GRB2*, *SNX5*, and *AKT1*, had a hazard ratio (HR) >1. Meanwhile, *PSD4* was associated with an HR <1.

**Figure 2:**
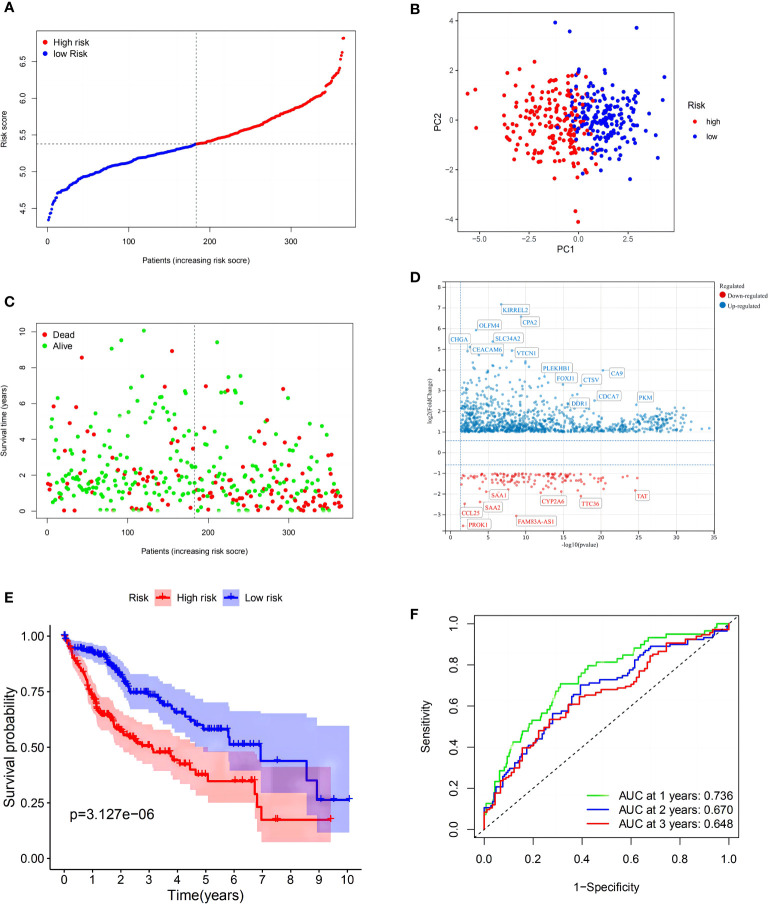
Construction of the risk signature in The Cancer Genome Atlas (TCGA) cohort. **(A)** Distribution of patients based on the risk score. **(B)** PCA plot for hepatocellular carcinomas (HCCs) based on the risk score. **(C)** The survival status for each patient (low-risk population: on the left side of the dotted line; high-risk population: on the right side of the dotted line). **(D)** Volcano plot showing the significant DEGs with FDR <0.05 and |log2FC| >1 between two macropinocytosis score groups. **(E)** Kaplan–Meier curves for OS of patients in the high- and low-risk groups (blue: low-risk group; red: high-risk group). **(F)** ROC curves showing the predictive efficiency of the risk score.

LASSO Cox regression was then used to generate a seven-gene signature model based on cross-validation with *GSK3B*, *AXIN1*, *RAC1*, *KEAP1*, *EHD1*, *GRB2*, and *SNX5*. The following formula was used to calculate the risk score: RS = 0.0606 * EXP (*GSK3B*) + 0.0326 * EXP (*AXIN1*) + 0.2757 * EXP (*RAC1*) + 0.2323 * EXP (*KEAP1*) + 0.0852 * EXP (*EHD1*) + 0.2713 * EXP (*GRB2*) + 0.3377 * EXP (*SNX5*). Using the med-cutoff score, the HCC samples were separated into the LRS and HRS groups (**Figure 2A**). According to PCA, the samples were well differentiated into the two clusters and distributed in discrete directions (**Figure 2B**). LRS patients had a longer survival time than those in the HRS group (*p* < 0.001) (**Figure 2C**). A volcano plot identified the significant DEMRGs in the two RS groups (**Figure 2D**). To determine the ability of the model to predict OS, Kaplan–Meier curves were developed to compare different survival years between the two groups. LRS HCC patients were found to have longer OS than HRS patients (*p* < 0.001) (**Figure 2E**). The RS-constructed predictive signature model was assessed using time-dependent ROC analysis, and the areas under the ROC curve (AUCs) were 0.736, 0.670, and 0.648 at 1, 2, and 3 years, respectively (**Figure 2F**). Considering that the AUC of 2 and 3 years is not high enough, this suggests that the survival prediction ability of the model for the TCGA cohort at these time points is not as good as that of 1 year, so we have analyzed the ROC curve of approximately 1 year (9 and 15 months). As we expected, the AUC of the three-time points in **Supplementary Figure 1** is at a good level. Perhaps, in the TCGA cohort, the model has the best prediction level at approximately 1 year, and this shows that the short-term prediction value of the model for the TCGA is more worthy of our attention.

**Figure 3:**
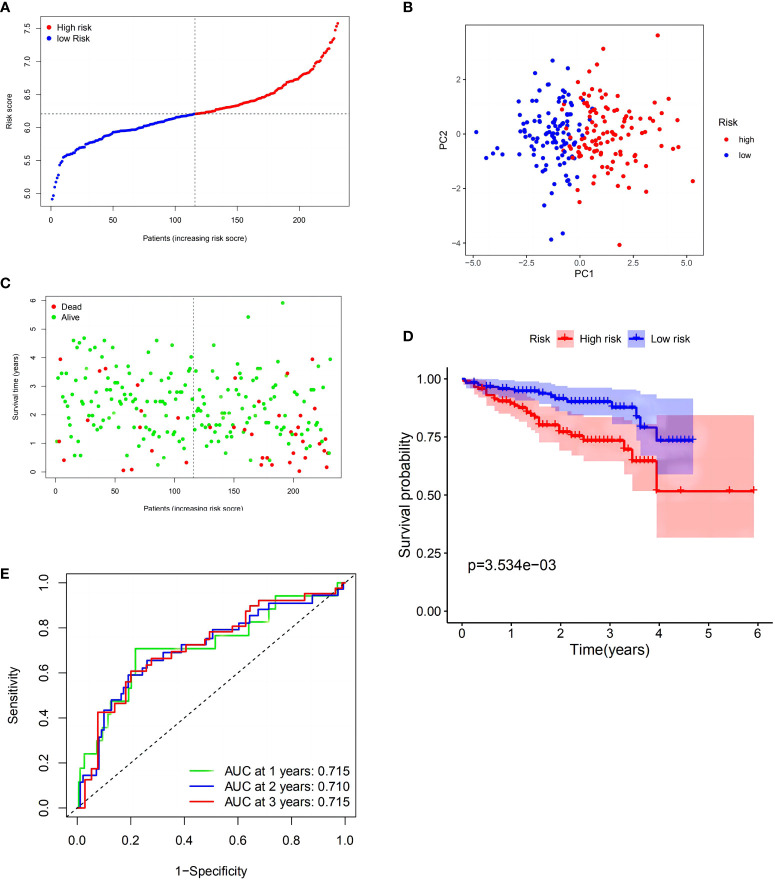
Validation of the risk model in the International Cancer Genome Consortium (ICGC) cohort. **(A)** Distribution of patients in the ICGC cohort based on the median risk score in the TCGA cohort. **(B)** PCA plot for HCCs. **(C)** Survival status for each patient (low-risk population: on the left side of the dotted line; high-risk population: on the right side of the dotted line). **(D)** Kaplan–Meier curves comparingOS between the low- and high-risk groups. **(E)** Time-dependent ROC curves for HCCs.

To verify these results, 273 HCC samples from the ICGC were also separated into two groups (**Figure 3A**). The PCA revealed identical results (**Figure 3B**). As described above, HCC samples in the LRS group had longer survival (*p* < 0.01) than those in the HRS group (**Figures 3C, D**). These findings indicated that the model also showed strong forecasting competence in the ICGC. The ICGC AUCs at 1-, 2-, and 3-year OS were 0.715, 0.710, and 0.715, respectively (**Figure 3E**). Interestingly, the model indicated that the validation cohort had a better survival rate and discrimination ability than the TCGA cohort. In the GEO validation cohort, we applied the same statistical method to validate our model, and the resulting figures are shown in **Supplementary Figure 2**, which have also achieved good results, providing support for the reliability of the model.

### The independent prognostic role of the MRPM

After constructing the MRPM for the HRS and LRS groups, we created a clinical information table of the patients from the TCGA, including THEIR age, gender, tumor grade, and TNM staging (the missing information was deleted). Analysis of clinical data revealed that a higher risk score was strongly correlated with both the TNM staging and tumor histologic grade (**Supplementary Table 1**). Meanwhile, univariate and multivariate Cox regression analyses of data from the TCGA showed that the tumor TNM staging (*p* < 0.001) and RS (*p* < 0.001) were prominent IPFs for HCC patients (**Figures 1D, E**).

### Model-based enrichment analysis

**Figure 4:**
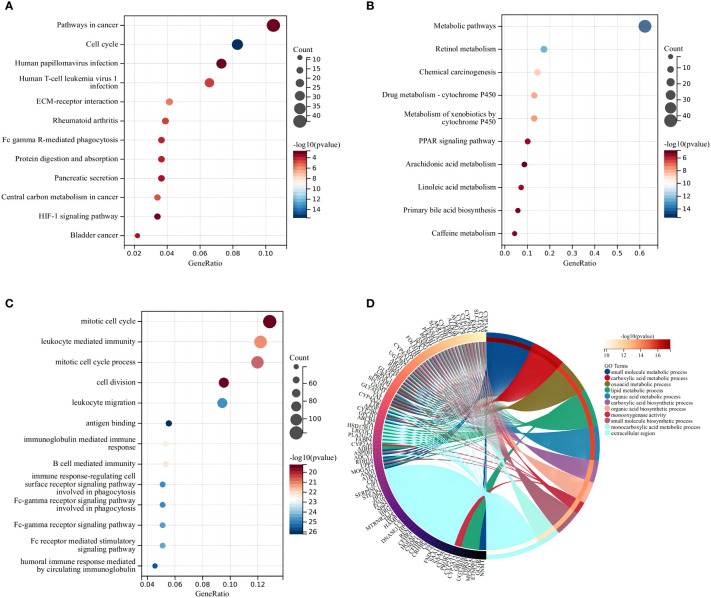
Functional analysis based on the differentially expressed genes between the two risk groups in the TCGA cohort. The larger bubble and the longer bar mean that more genes were enriched, and the increasing depth of red means that the differences were more obvious. **(A)** Bubble graph for the high expression of KEGG pathways in the TCGA cohort. **(B)** Bubble graph for the low expression of KEGG pathways in the TCGA cohort. **(C)** Bubble graph for the high expression of GO enrichment in the TCGA cohort. **(D)** Circular plot for the low expression of GO enrichment in the TCGA cohort.

KEGG indicated that the differentially expressed DEMRGs were chiefly mapped to “pathways in cancer,” “cell cycle,” and “human T-cell leukemia virus-1 infection” (**Figures 4A, B**), while GO functional analyses found that the DEMRGs were enriched in “mitotic cell cycle,” “leukocyte-mediated immunity,” and “cell division” (**Figures 4C, D**). These findings indicated that differences in the OS of patients in each risk group may be related to differences in the immune response or cell cycle.

### Immune status of HCC patients in each subgroup

**Figure 5:**
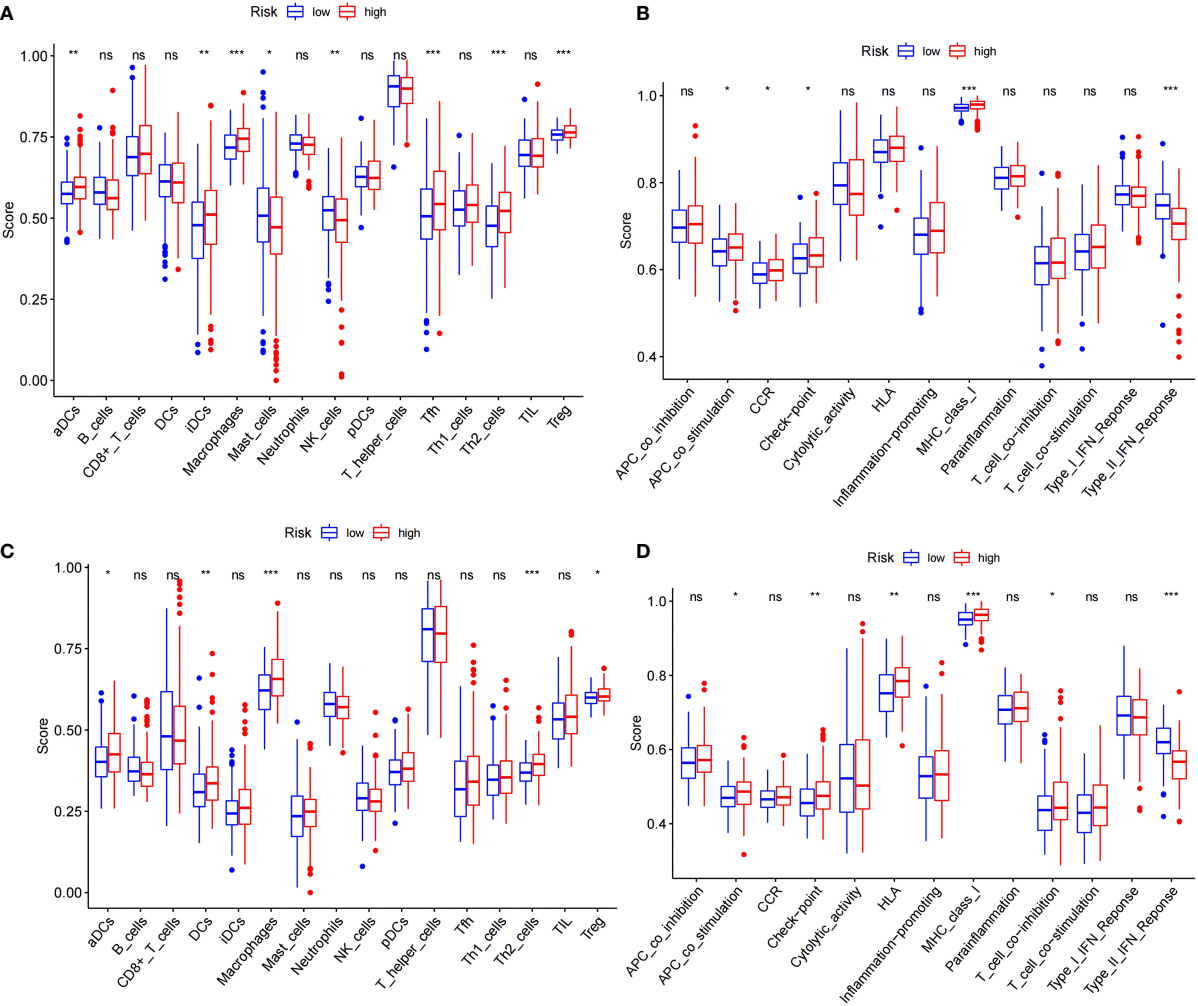
Comparison of the ssGSEA scores for immune cells and immune pathways. **(A, B)** Comparison of the enrichment scores of 16 types of immune cells and 13 immune-related pathways between the low- (blue box) and high-risk (red box) groups in the TCGA cohort. **(C, D)** Comparison of tumor immunity between the low- (blue box) and high-risk (red box) groups in the ICGC cohort. *p < 0.05; **p < 0.01; ***p < 0.001. ns: No significant difference.

A more in-depth functional analysis of the type of infiltrating immunocytes and the activity of the immunological pathways in the two databases was conducted using ssGSEA. In the TCGA (**Figure 5A**), the LRS patients had lower levels of immunocyte enrichment, macrophages, and Tfh cells than the HRS patients. While the type II interferon response was stronger in the LRS group, other pathways, including MHC class I, checkpoint, and antigen-presenting cell (APC) co-stimulation, were more active in the HRS group (**Figure 5B**). Meanwhile, in the ICGC cohort, macrophage, Th2 cell, and dendritic cell (DC) infiltration were lower in the LRS group. Except for the type II interferon response, other immunological pathways, such as MHC class I, human leukocyte antigen (HLA), checkpoint, and APC co-stimulation, were also lower in the LRS group than in the HRS group (**Figures 5C, D**). These results were consistent with the TCGA cohort, further supporting the reliability of the results and suggesting that the poorer survival of HRS HCC patients may result, in part, from an immunosuppressive microenvironment.

**Figure 6:**
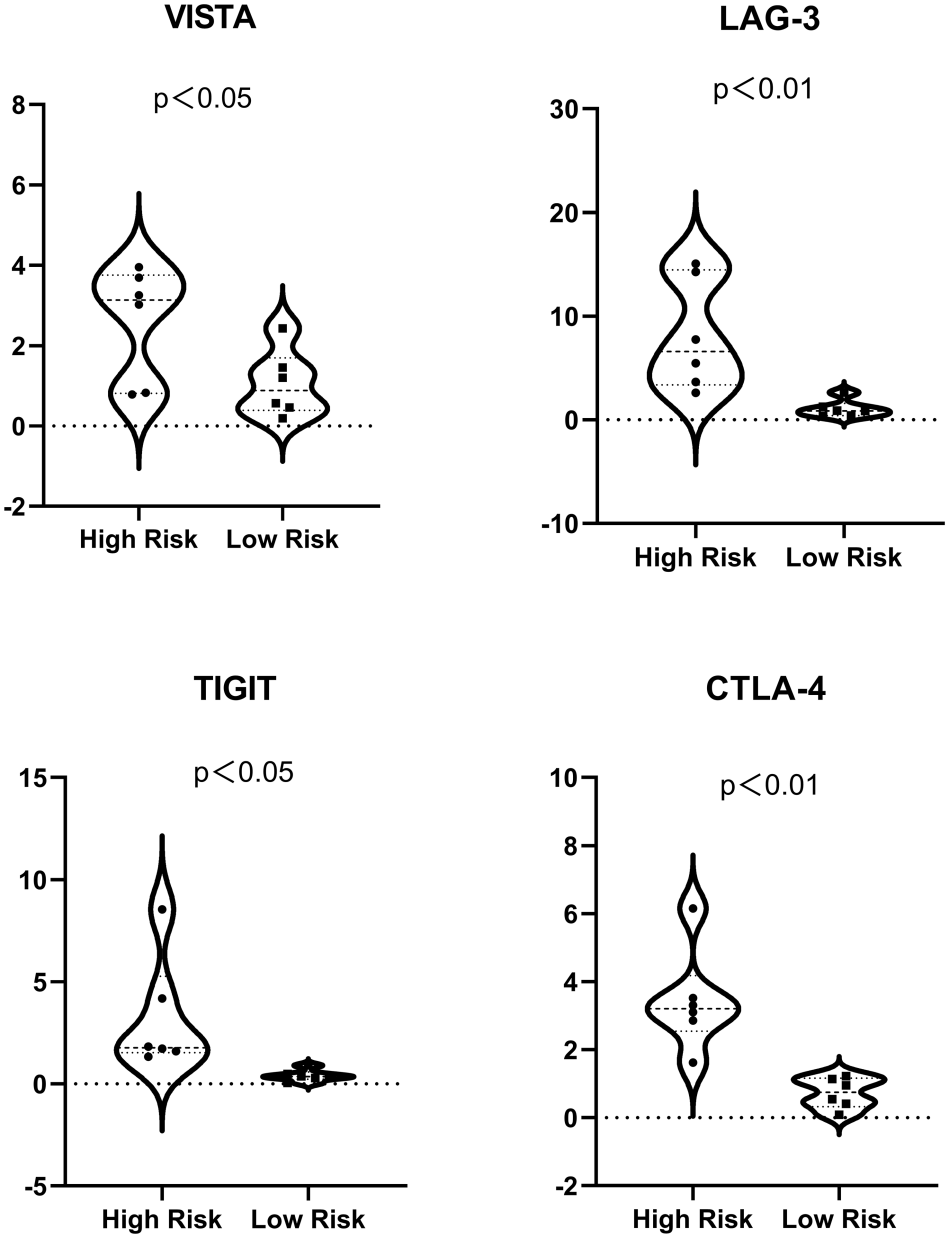
Expression of immune checkpoints (VISTA, LAG-3, TIGIT, and CTLA-4) in the high- and low-risk samples. For ease of reading, the unit of the vertical axis is 1/1,000.

Immune checkpoint gene expression was also compared between the HRS and LRS groups since the expression levels of immune checkpoints are important indicators of the success of immunotherapy for multiple cancers (**Figure 6**; **Supplementary List 2**). The expression of cytotoxic T lymphocyte-associated protein 4 (CTLA-4), lymphocyte activation gene-3 (LAG-3), the V-domain Ig suppressor of T-cell activation (VISTA), and the T-cell immunoreceptor with Ig and ITIM domains (TIGIT) were slightly lower in the LRS samples than in the HRS samples (*p* < 0.05), suggesting that the reduced survival of the HRS group may be associated with an immunosuppressive microenvironment and supporting the verification results described above.

## Discussion

In recent years, progress in sequencing technology has exposed the genetic landscape of HCC (6) and shown that dysregulated gene expression is associated with several biological processes, including signal transduction, nutrient absorption, and antigen uptake. These involve different pathways, of which MPC dysregulation is closely linked with the growth, immune response, and death of HCC cells (7). Recent studies have identified a correlation between HCC and MPC.

A recent study found that, through the HIF/EHD2 pathway, hypoxia induces extensive HCC cell MPC, allowing the tumor to acquire exocytic nutrients and prolong survival (7). Hypoxia-inducible factor (HIF) mediates HCC cell adaptation and spread. Eps15 homology-domain containing protein 2 (EHD2), a membrane-ruffling transcription factor, activates HIF-1 and initiates MPC. Knocking out HIF-1 or EHD2 inhibits hypoxia-induced MPC and prevents hypoxic HCC cells from salvaging nutrients required for cell growth. In mice, HCC development and MPC are inhibited by EHD2 somatic or germline deletion or by using HIF-1 or MPC inhibitors. These findings provide a novel direction for liver cancer research. Coincidentally, EHD1 was one of the seven DEMRGs selected in the current study. All four EHD family members, EHD1–EHD4, are dynamin-related ATPases involved in regulating specific endocytic transport steps through direct and indirect binding to actin filaments (17). The KEGG analyses conducted by our study showed that the differential risk genes we obtained were also considerably enriched in the HIF-1 pathway. These findings make it clear that MPC plays an important role in regulating tumor growth, dissemination, and antitumor responses. Strategies aimed at modifying MPC status in HCC patients may greatly improve their prognosis.

A recent study by Byun et al. found that sorafenib induces MPC, which mitigates sorafenib−induced ferroptosis and confers drug resistance (18). Sorafenib, a multiple tyrosine and downstream serine/threonine kinase inhibitor that triggers apoptosis and prevents angiogenesis and cancer cell proliferation, was the first targeted drug for HCC to be approved worldwide (19). In HCC samples, sorafenib was shown to inactivate mitochondrial function through PI3K–RAC1–PAK1 signaling and to amplify HCC cell MPC. Ferroptosis, a form of cell death that occurs through the lethal accretion of lipid-based reactive oxygen species, is closely associated with the development of HCC (20, 21). In recent years, medications have been developed that specifically target HCC cell ferroptosis (22). However, MPC inhibits ferroptosis by restocking depleted endocellular cysteine, causing HCC cells to develop resistance to sorafenib (18). MPC differs from other types of endocytosis in part due to its susceptibility to inhibitors of Na^+^/H^+^ exchange. Amiloride, a guanidinium-containing pyrazine derivative, is extensively used as a sodium–hydrogen exchanger (NHE) inhibitor. NHEs are membrane transporters that mediate the electroneutral exchange of hydrogen efflux and sodium influx to regulate intracellular pH (23). NHE inhibition reduces cytosolic pH, thus causing sensitive changes in GTPases that promote actin remodeling and selectively block MPC. Thus, targeting MPC may prevent the development of sorafenib resistance and improve the efficacy of this drug against HCC.

For patients with advanced HCC, current targeted therapeutic drugs such as sorafenib and lenvatinib showed only limited survival benefits with many potential side effects (24). Just recently, after the great success of the IMbrave150 clinical trial in 2019, the relevant research on various immune checkpoint inhibitors (ICIs) has attracted the attention of researchers (24). The complex immune microenvironment of HCC provides further potential advantages for ICI-based therapies in HCC.

As we all know, after anti-programmed cell death protein 1/programmed cell death ligand 1 (PD-1/PD-L1) got satisfying results in most cancers, CTLA-4 has also made breakthroughs in the immunotherapy of various tumors. Engagement of LAG-3 by its ligands can contribute to the immune escape of tumor cells by mediating downstream signaling to affect T-cell immune activity. Therefore, blockages of the LAG-3-related inhibitory signaling are promising for the development of new therapies (25). VISTA can blunt the activity of antitumor cells and strengthen the power of protumor cells. After inhibition of other immune checkpoints, VISTA’s expression profile will increase, which indicates that VISTA could constitute a new complementary target in immunotherapy (26). TIGIT is mainly expressed on tumor-infiltrating cytotoxic T cells, NK cells, etc., contributing to local suppression of immune surveillance *via* interacting with CD155. Several preclinical studies have supported the use of TIGIT blockade for the treatment of some advanced malignant tumors (27).

Our model classifies HCC patients into HRS and LRS groups according to the relative expression of seven genes. The results show that there is a significant difference in the expression of CTLA-4/LAG-3/TIGIT/VISTA between the two groups, suggesting that the mechanism of the effect of macropinocytosis on the progression of HCC may be related to these immune checkpoints, which are promising targets for antitumor immunotherapy. Therefore, our model can provide new ideas for the innovation of immunotherapy regimens. We can conduct clinical trials in the future when the conditions are mature. In future studies, a comparison of the antitumor effect of two regimens (immunodrugs alone and immunodrugs combined with macropinocytosis inhibitors) in HCC patients may provide new insights.

It is well known that immunotherapy brings hope for improving the survival rate of HCC patients, but at the same time, a relative proportion of patients will develop drug resistance during immunotherapy, which has a significant impact on the prognosis of these patients. Among them, the drug resistance of HCC patients is mainly divided into primary drug resistance without initial response and secondary drug resistance after the initial control. At present, studies have found that the drug resistance of immunotherapy is related to a variety of potential mechanisms, including downregulation of tumor immunogenic antigen expression or impaired antigen presentation, mutation or deletion of genes related to signaling pathway (interferon-γ, WNT/β-catenin, etc.), immunosuppressive tumor microenvironment (TME), and generation of alternative immune checkpoints. Among them, TIM-3, LAG-3, and TIGIT are currently the hottest alternative immune checkpoint targets. Therefore, we associate that our risk model may be used to monitor the drug resistance of HCC patients after immunotherapy and evaluate the severity of drug resistance. After all, we have not found biomarkers that can well predict drug resistance reactions.

The current study has some limitations. First, some variables, including ethnicity, HCC cause, patient-related imaging data, and serum indicators, had high levels of missing data so they were not included in the datasets. The cause of HCC differs by geographic region and race, requiring different methods of prognosis and treatment (28). The specificity and sensitivity of prognostic models are improved with more imaging data and markers (16). Second, although the model we have constructed has been well verified in multiple public databases, there are some differences in different databases, such as the reliability and the best scope of application of the model. We should deepen our understanding of the molecular function of MPC and explore more genes that may be related to MPC in order to improve the reliability and applicability of MRPM. Thus, future studies should verify the findings of the current study with additional risk and associated genes to expand the current molecular understanding of MPC. A prospective study is also needed to validate the results of the current retrospective study (15).

## Conclusion

This study used a panel of seven DEMRGs and clinical characteristics to build a prognostic model and validated the findings with data from a second database. The findings demonstrated strong discriminative power to predict HCC patient survival, showing promising prediction value and the ability to serve as a reference for the clinical stratification of HCC. The findings also partially confirmed the relevancy between the model and immunity, informing the development of new immunotherapeutic strategies to treat this disease (14).

## Data availability statement

The datasets presented in this study can be found in online repositories. The names of the repository/repositories and accession number(s) can be found in the article/**Supplementary Material**.

## Ethics statement

Written informed consent was obtained from the individual(s) for the publication of any potentially identifiable images or data included in this article.

## Author contributions

All authors contributed to the conception and design of the study. Data collection and analysis were performed by XD. TY reviewed the literature and prepared the material. The first draft of the manuscript was written by XD. All authors commented on the subsequent versions of the manuscript. All authors contributed to the article and approved the submitted version.

## References

[1] Villanueva A. Hepatocellular carcinoma. N Engl J Med (2019) 380:1450–62. doi: 10.1056/NEJMra1713263 30970190

[2] Greten TF Lai CW Li G Staveley-O'Carroll KF. Targeted and immunebased therapies for hepatocellular carcinoma. Gastroenterol (2019) 156:510–24. doi: 10.1053/j.gastro.2018.09.051 PMC6340758 30287171

[3] Gao Q Wang XY Zhou J Fan J. Multiple carcinogenesis contributes to the heterogeneity of HCC. Nat Rev Gastroenterol Hepatol (2015) 12(1):13. doi: 10.1038/nrgastro.2014.6-c1 25421581

[4] Nakagawa S Wei L Song WM Higashi T Ghoshal S Precision Liver Cancer Prevention Consortium. Molecular liver cancer prevention in cirrhosis by organ transcriptome analysis and lysophosphatidic acid pathway inhibition. Cancer Cell (2016) 30(6):879–90. doi: 10.1016/j.ccell.2016.11.004 PMC5161110 27960085

[5] Petrizzo A Mauriello A Tornesello ML Buonaguro FM Tagliamonte M Buonaguro L. Cellular prognostic markers in hepatitis-related hepatocellular carcinoma. Infect Agent Cancer (2018) 13:10. doi: 10.1186/s13027-018-0183-8 29599818 PMC5870199

[6] Zhang MS Cui JD Lee D Yuen VW-H Chiu DK-C Goh CC. Regulated portals of entry into the cell. Nature (2003) 422:37–44. doi: 10.1038/nature01451 12621426

[7] Misty SZ Jane DC Lee D Buonaguro FM Tagliamonte M Buonaguro L. Hypoxia-induced macropinocytosis represents a metabolic route for liver cancer. Nat Commun (2022) 13(1):954. doi: 10.1038/s41467-022-28618-9 35177645 PMC8854584

[8] Finicle BT Jayashankar V Edinger AL. Nutrient scavenging in cancer. Nat Rev Cancer (2018) 18(10):619–33. doi: 10.1038/s41568-018-0048-x 30097614

[9] Kamphorst JJ Nofal M Commisso C Hackett SR Lu W Grabocka E. Human pancreatic cancer tumors are nutrient poor and tumor cells actively scavenge extracellular protein. Cancer Res (2015) 75:544–53. doi: 10.1158/0008-5472.CAN-14-2211 PMC4316379 25644265

[10] Jayashankar V Edinger AL. Macropinocytosis confers resistance to therapies targeting cancer anabolism. Nat Commun (2020) 11(1):1121. doi: 10.1038/s41467-020-14928-3 32111826 PMC7048872

[11] Kim SM Nguyen TT Ravi A Kubiniok P Finicle BT Jayashankar V. PTEN deficiency and AMPK activation promote nutrient scavenging and anabolism in prostate cancer cells. Cancer Discov (2018) 8(7):866–83. doi: 10.1158/2159-8290.CD-17-1215 PMC6030497 29572236

[12] Kay RR. Macropinocytosis: Biology and mechanisms. Cells Dev (2021) 168:203713. doi: 10.1016/j.cdev.2021.203713 34175511

[13] Song S Zhang Y Ding T Ji N Zhao H. The dual role of macropinocytosis in cancers: Promoting growth and inducing methuosis to participate in anticancer therapies as targets. Front Oncol (2021) 10:570108. doi: 10.3389/fonc.2020.570108 33542897 PMC7851083

[14] Deng M Sun S Zhao R Guan R Zhang Z Li S. The pyroptosis-related gene signature predicts prognosis and indicates immune activity in hepatocellular carcinoma. Mol Med (2022) 28(1):16. doi: 10.1186/s10020-022-00445-0 35123387 PMC8818170

[15] Deng T Hu B Jin C Tong Y Zhao J Shi Z. A novel ferroptosis phenotype-related clinical-molecular prognostic signature for hepatocellular carcinoma. J Cell Mol Med (2021) 25(14):6618–33. doi: 10.1111/jcmm.16666 PMC8278110 34085405

[16] Wan S Lei Y Li M Wu B. A prognostic model for hepatocellular carcinoma patients based on signature ferroptosis-related genes. Hepatol Int (2022) 16(1):112–24. doi: 10.1007/s12072-021-10248-w 34449009

[17] Naslavsky N Caplan S. EHD proteins: key conductors of endocytic transport. Trends Cell Biol (2011) 21:122–31. doi: 10.1016/j.tcb.2010.10.003 PMC3052690 21067929

[18] Byun J-K Lee S Kang GW Lee YR Park SY Song IS. Macropinocytosis is an alternative pathway of cysteine acquisition and mitigates sorafenib-induced ferroptosis in hepatocellular carcinoma. J Exp Clin Cancer Res (2022) 41(1):98. doi: 10.1186/s13046-022-02296-3 35287706 PMC8919615

[19] Llovet JM Ricci S Mazzaferro V Hilgard P Gane E Blanc JF. Sorafenib in advanced hepatocellular carcinoma. N Engl J Med (2008) 359(4):378–90. doi: 10.1056/NEJMoa0708857 18650514

[20] Yang WS SriRamaratnam R Welsch ME Shimada K Skouta R Viswanathan VS. Regulation of ferroptotic cancer cell death by GPX4. Cell (2014) 156:317–31. doi: 10.1016/j.cell.2013.12.010 PMC4076414 24439385

[21] Dixon SJ Lemberg KM Lamprecht MR Skouta R Zaitsev EM Gleason CE. Ferroptosis: an iron-dependent form of nonapoptotic cell death. Cell (2012) 149:1060–72. doi: 10.1016/j.cell.2012.03.042 PMC3367386 22632970

[22] Sun X Ou Z Chen R Niu X Chen D Kang R. Activation of the p62-Keap1-NRF2 pathway protects against ferroptosis in hepatocellular carcinoma cells. Hepatol (2016) 63:173–84. doi: 10.1002/hep.28251 PMC4688087 26403645

[23] Koivusalo M Welch C Hayashi H Scott CC Kim M Alexander T. Amiloride inhibits macropinocytosis by lowering submembranous pH and preventing Rac1 and Cdc42 signaling. J Cell Biol (2010) 188(4):547–63. doi: 10.1083/jcb.200908086 PMC2828922 20156964

[24] Liu JKH Irvine AF Jones RL Samson A. Immunotherapies for hepatocellular carcinoma. Cancer Med (2022) 11(3):571–91. doi: 10.1002/cam4.4468 PMC8817091 34953051

[25] Tian J Liu Y Zhang T Yue L Xiao Y Guo C. LAG-3 is a promising inhibitory immune checkpoint for antitumor immunotherapy. Expert Rev Anticancer Ther (2022) 22(3):289–96. doi: 10.1080/14737140.2022.2039124 35132925

[26] Mortezaee K Majidpoor J Najafi S. VISTA immune regulatory effects in bypassing cancer immunotherapy: Updated. Life Sci (2022) 310:121083. doi: 10.1016/j.lfs.2022.121083 36265568

[27] Ge Z Peppelenbosch MP Sprengers D Kwekkeboom J. TIGIT, the next step towards successful combination immune checkpoint therapy in cancer. Front Immunol (2021) 12:699895. doi: 10.3389/fimmu.2021.699895 34367161 PMC8339559

[28] Lee BP Vittinghoff E Dodge JL Cullaro G Terrault NA. National trends and long-term outcomes of liver transplant for alcohol-associated liver disease in the united states. JAMA Intern Med (2019) 179:340–8. doi: 10.1001/jamainternmed.2018.6536 PMC6439700 30667468

