# Supplementary figures and images for: A macropinocytosis-related gene signature predicts the prognosis and immune microenvironment in hepatocellular carcinoma
Source: Front Oncol. 2023 Mar 30;13:1143013. doi: 10.3389/fonc.2023.1143013 (PMC10097907; doi:10.3389/fonc.2023.1143013)

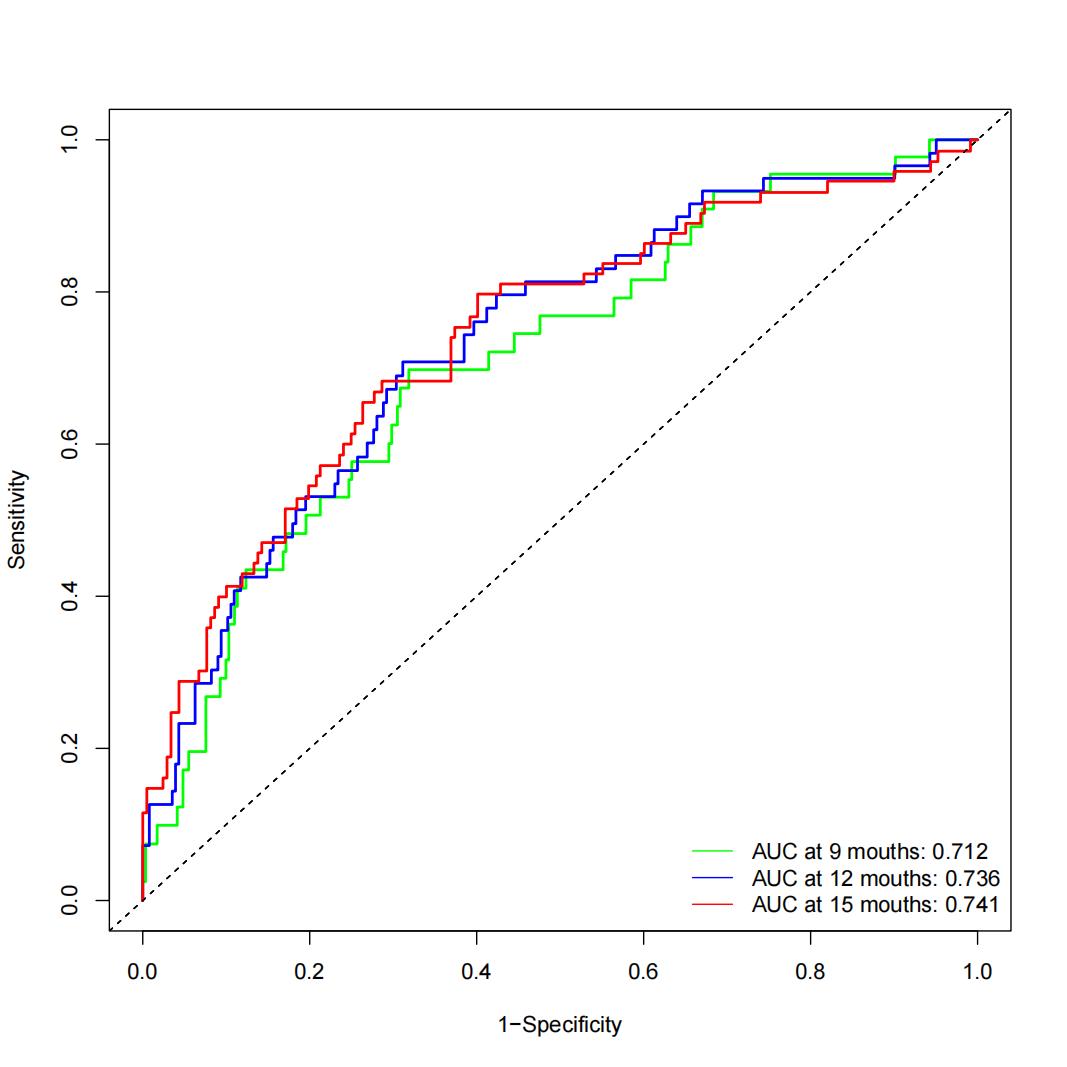

Supplement: Supplementary file 1 [file Image_1.jpeg]

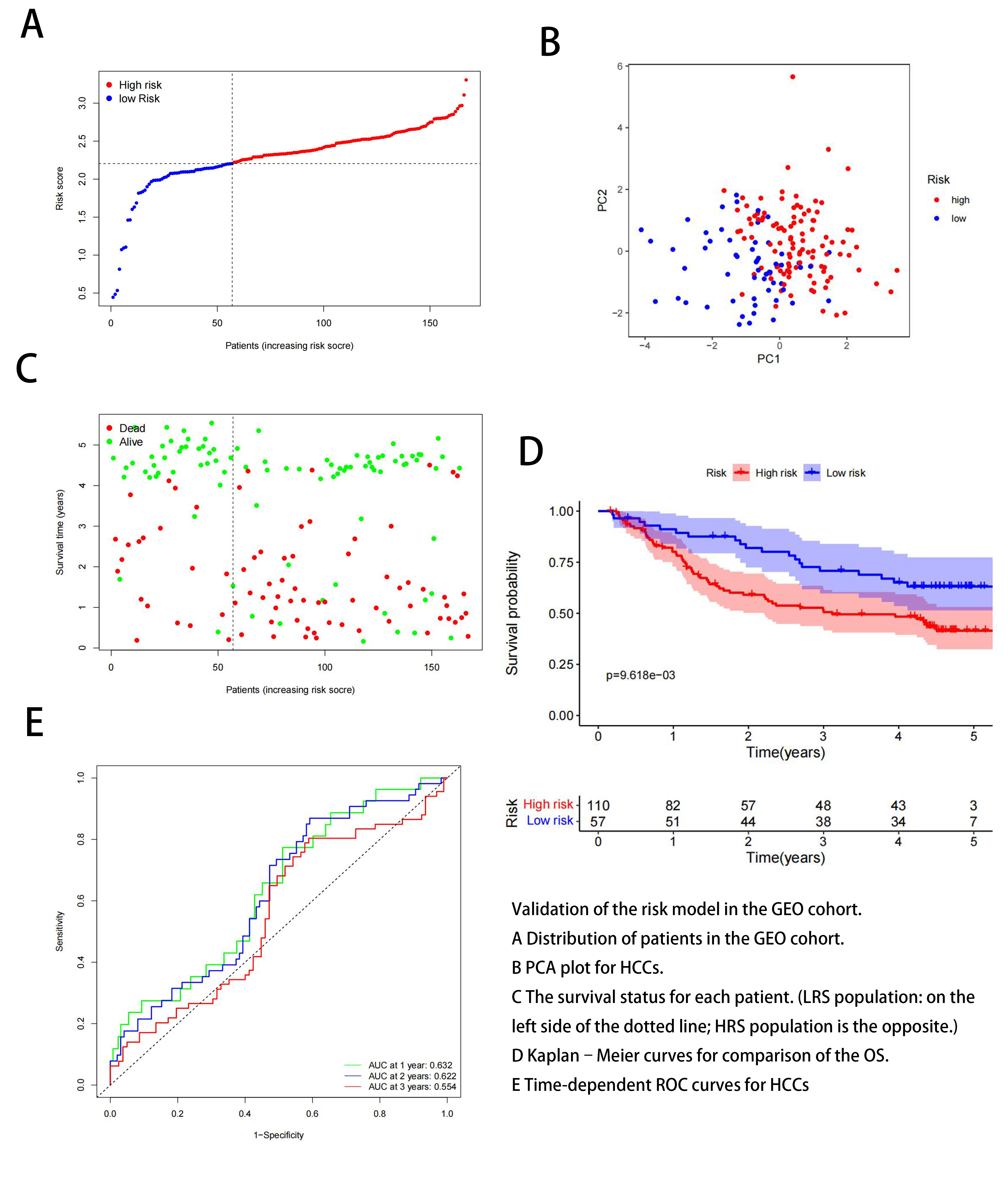

Supplement: Supplementary file 2 [file Image_2.png]

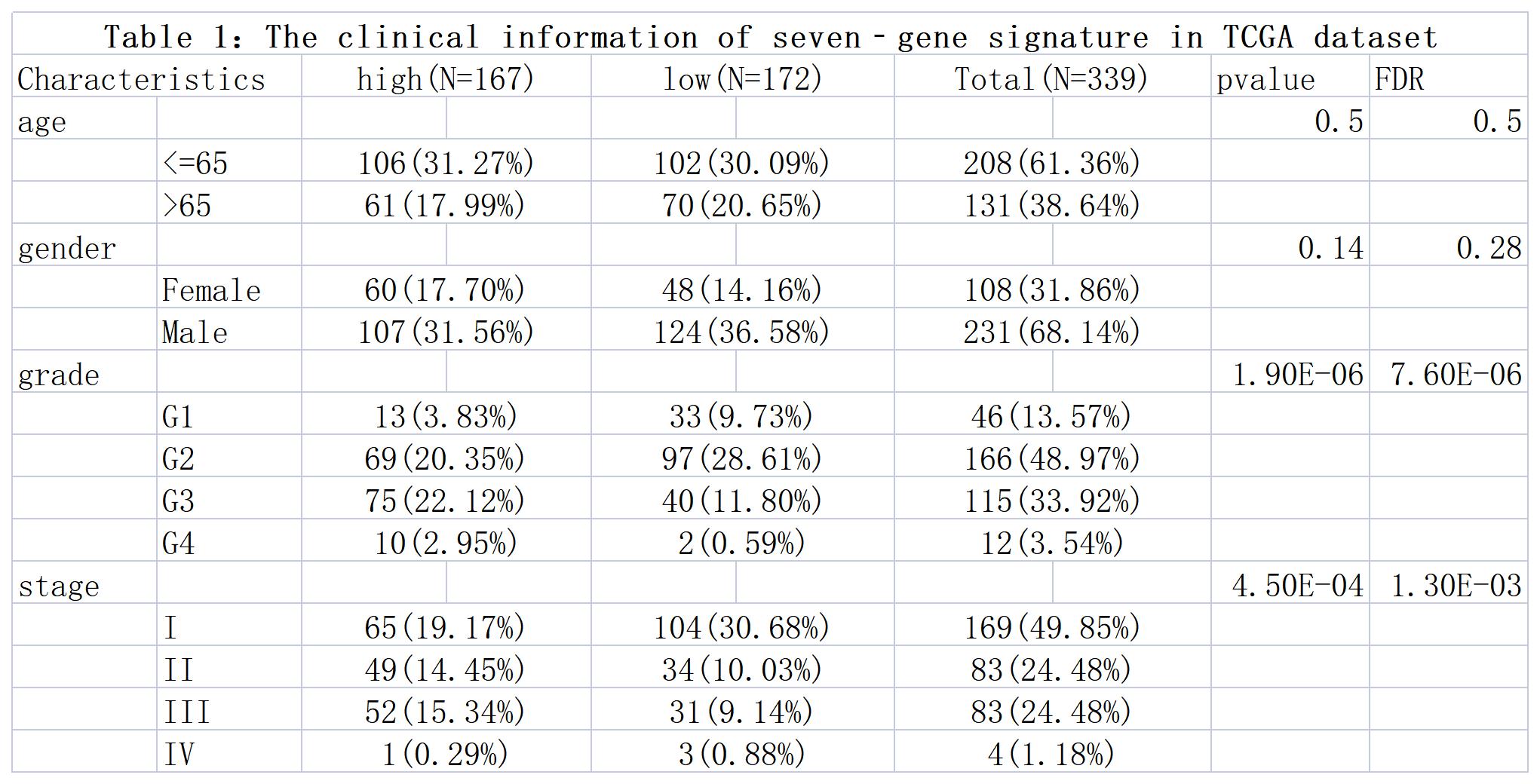

Supplement: Supplementary file 3 [file Image_3.jpeg]
