# Supplementary material for: A macropinocytosis-related gene signature predicts the prognosis and immune microenvironment in hepatocellular carcinoma
Source: Front Oncol. 2023 Mar 30;13:1143013. doi: 10.3389/fonc.2023.1143013 (PMC10097907; doi:10.3389/fonc.2023.1143013)
Supplement: Supplementary file 5 [file Table_2.doc]

Model related genes

GSK3B

Sequence (5' -> 3') Length Tm Location

Forward Primer AGACGCTCCCTGTGATTTATGT 22 61.2 452-473

Reverse Primer CCGATGGCAGATTCCAAAGG 20 60.8 540-521

AXIN1

Sequence (5' -> 3') Length Tm Location

Forward Primer GGTTTCCCCTTGGACCTCG 19 62.0 19-37

Reverse Primer CCGTCGAAGTCTCACCTTTAATG 23 60.9 175-153

RAC1

Sequence (5' -> 3') Length Tm Location

tacgccccctatcctatccg

atgggagtgttgggacagtg

KEAP1

Sequence (5' -> 3') Length Tm Location

Forward Primer CTGGAGGATCATACCAAGCAGG 22 61.9 166-187

Reverse Primer GGATACCCTCAATGGACACCAC 22 62.1 385-364

EHD1

Sequence (5' -> 3') Length Tm Location

Tggacacggtggatgacatg

cgttcatggtgccgtcaaag

GRB2

Sequence (5' -> 3') Length Tm Location

Forward Primer CTGGGTGGTGAAGTTCAATTCT 22 60.2 360-381

Reverse Primer GTTCTATGTCCCGCAGGAATATC 23 60.3 457-435

SNX5

Sequence (5' -> 3') Length Tm Location

Forward Primer CAGAGCCCAGAGTTTTCTGTTAC 23 60.8 175-197

Reverse Primer CCCAGCATAGTCTGTTGTTTCA 22 60.2 261-240

Immune checkpoint

CTLA4

Sequence (5' -> 3') Length Tm Location

Forward Primer CATGATGGGGAATGAGTTGACC 22 60.7 267-288

Reverse Primer TCAGTCCTTGGATAGTGAGGTTC 23 60.8 358-336

LAG3 CD223

Tggcgactttacccttcgac

Tgtgacagtggcattgagct

TIGIT

Sequence (5' -> 3') Length Tm Location

Atgggacgtacactgggaga

Ttctagtcaacgcgaccacc

IDO1

Sequence (5' -> 3') Length Tm Location

Forward Primer TCTCATTTCGTGATGGAGACTGC 23 62.2 455-477

Reverse Primer GTGTCCCGTTCTTGCATTTGC 21 62.6 584-564

TDO2

Sequence (5' -> 3') Length Tm Location

Forward Primer AAGGTTGTTTCTCGGATGCAC 21 60.8 328-348

Reverse Primer TGTCATCGTCTCCAGAATGGAA 22 60.8 408-387

PDCD1 PD1

Sequence (5' -> 3') Length Tm Location

Forward Primer CCAGGATGGTTCTTAGACTCCC 22 61.2 61-82

Reverse Primer TTTAGCACGAAGCTCTCCGAT 21 61.3 197-177

HHLA2

Sequence (5' -> 3') Length Tm Location

Forward Primer TACAAAGGCAGTGACCATTTGG 22 60.8 223-244

Reverse Primer AGGTGTAAATTCCTTCGTCCAGA 23 61.0 361-339

VISTA VSIR

Sequence (5' -> 3') Length Tm Location

Forward Primer ACGCCGTATTCCCTGTATGTC 21 61.7 109-129

Reverse Primer TTGTAGAAGGTCACATCGTGC 21 60.0 209-189

TIM-3 HAVCR2

Sequence (5' -> 3') Length Tm Location

Forward Primer CTGCTGCTACTACTTACAAGGTC 23 60.1 40-62

Reverse Primer GCAGGGCAGATAGGCATTCT 20 61.8 114-95

BTLA CD272

Sequence (5' -> 3') Length Tm Location

Forward Primer CATCTTAGCAGGAGATCCCTTTG 23 60.2 141-163

Reverse Primer GACCCATTGTCATTAGGAAGCA 22 60.0 335-314
